# Supplementary material for: Randomized controlled trial of an Internet-of-Medical-Things device for patient-guided anorectal biofeedback therapy
Source: PLoS One. 2024 Sep 27;19(9):e0311134. doi: 10.1371/journal.pone.0311134 (PMC11432836; doi:10.1371/journal.pone.0311134)
Supplement: S1 File — (DOCX) [file pone.0311134.s001.docx]

**COMPARISON OF ANORECTAL BIOFEEDBACK THERAPY DELIVERED BY HOME-BASED DEVICE WITH CLINIC-BASED ANORECTAL MANOMETRY IN PATIENTS WITH FUNCTIONAL DEFECATION DISORDERS**

**Sponsor**

School of Medicine, Western Sydney University

Ainsworth Building 30, Goldsmith Avenue, Campbelltown 2560

**Principle Investigator**

Dr Vincent Ho | Gastroenterologist | Department of Gastroenterology, Campbelltown Hospital, Campbelltown NSW 2560

**Chief Investigator/Researcher**

Dr Jerry Zhou | Lecturer | School of Medicine, Western Sydney University, Campbelltown NSW 2560

**Associate/Co-Investigator** **(Site 1: Camden Hospital)**

Billie McHutchison | Specialist Nurse | GI Motility Clinic, Camden Hospital, Camden NSW

**Co-ordinating Principal Investigator (Site 2: Royal Prince Alfred Hospital)**

Kheng-Seong Ng | Colorectal Surgeon | Royal Prince Alfred Hospital

**Chief Investigator/Researcher (Site 2: Royal Prince Alfred Hospital)**

Dr Vicki Patton | Advanced GI Surgery Unit | Royal Prince Alfred Hospital

**TABLE OF CONTENTS**

PROTOCOL TITLE 1

SPONSOR 1

INVESTIGATORS________________________________________________________1

1. SUMMARY 3

2. BACKGROUND AND RATIONALE 3

3. STUDY AIMS/OBJECTIVES 4

4. PARTICIPATING SITES 4

5. STUDY DESIGN 5

5.1 STUDY TYPE 5

5.2 EXPECTED STUDY DURATION 5

5.3 DATA SOURCE AND POPULATION 5

5.4 RECRUITMENT AND SCREENING 6

5.5 INCLUSION CRITERIA 6

5.6 EXCLUSION CRITERIA 6

5.7 CONSENT PROCESS 6

5.8 STUDY PROCEDURES 6

7. ETHICAL CONSIDERATIONS 9

7.1 STUDY PROCEDURE BENEFITS 9

7.2 STUDY PROCEDURE RISKS 9

7.2.1 COVID-19 CONSIDERATIONS_________________________________________9

7.3 CONFIDENTIALITY AND PRIVACY 10

7.4 DATA STORAGE AND RECORD RETENTION 10

8. CONFLICT OF INTEREST 10

9. FUNDING 10

10. RESEARCH OUTCOMES 10

11. REFERENCES 11

**1. SUMMARY**

| **Study Title**: Comparison of anorectal biofeedback therapy delivered by home-based device with clinic-based anorectal manometry in patients with functional defecation disorders  **Aim:** To compare the treatment efficacy of anorectal biofeedback (clinical indicators and physiological parameters) performed with a novel home-based device with conventional therapies in patients with functional faecal incontinence.  **Site Location:**   1. GI Motility Clinic, Camden Hospital, NSW 2570 2. Advanced GI Surgery Unit, Royal Prince Alfred Hospital, NSW 2050   **Planned sample size:**   - Site 1: 40 participants (20 home-based with device, 20 clinic-based manometry) - Site 2: 80 participants (40 home-based with device, 40 clinic-based manometry)   **Inclusion criteria:** Males and females 18 – 65 years old, able to comprehend study and provide written consent. Diagnosed with a functional faecal incontinence and recommended for anorectal biofeedback therapy.  **Study procedures:**  A pilot feasibility study consisting of 40 participants will randomly assigned therapy with the novel device or anorectal manometry (conventional treatment). The conventional treatment group will receive 6 clinical training sessions with standard manometry (30 – 45 minutes each). While the test group will use the novel device at the clinic for 6 supervised training sessions, they may take the device home and continue training in between clinic sessions.  Upon completion of the feasibility study, a larger scale study (n=80) will be conducted. The test group will only receive one training session with the novel device and required to complete the rest of their training unsupervised at home.  **Study duration:** 3-years (36 months) |
| --- |

**2. BACKGROUND AND RATIONALE**

Anorectal biofeedback (BF) is a first-line rehabilitation therapy for the treatment of anorectal muscle disorders [1, 2]. These disorders cause 33% of chronic constipation and 60% of faecal incontinence [3, 4], two of the most prevalent gastrointestinal disorders, affecting 16% of the global population [5, 6]. BF therapy uses instrument-based “operant conditioning” (learning through repetition and reinforcement) to restore the normal muscle patterns for defecation. BF therapy is recommended by several consensus groups including the American and European Neurogastroenterology and Motility Societies [7]. Randomized clinical trials demonstrate BF efficacy of 75-89% in long-term symptom improvements [8, 9], and 83% reported improvements to quality-of-life [10]. Effective BF therapy demands regular attendance to a clinic over the treatment period of 6–8 weeks. Each session involves inserting a probe into the patient’s anal canal, which is then connected to a display for real-time visual feedback of muscle activities. Despite its benefits, BF is limited to few specialized clinics in tertiary hospital due to these challenges:

1. **Rising medical cost:** The high cost of equipment and technical staff required to deliver BF makes it difficult to establish clinics in regional and community hospitals.
2. **Time-consuming:** Average BF therapy involves the 6 weekly sessions and follow-up sessions after 6 and 12 months. Despite demand, clinics are limited by the number of patients they can treatment at one time.
3. **Adherence:** Difficulties accessing BF and the relative invasive nature of the procedure means fewer than 30% of patients are able to adhere and complete their prescribed treatment.

Anorectal manometry probes are the gold standard instrument for the diagnosis of functional anorectal disorders. Highly accurate sensors provide continuous and dynamic spatiotemporal mapping of anorectal pressures. Although this level of high fidelity data is not required for anorectal BF training, most clinics use the same manometry system due to ease of access [13]. However, these diagnostic systems are not well suited for BF therapy due to high setup and ongoing costs ($15K - $75K USD per system; $100 - $300 USD in consumables per use). In addition, the highly detailed data provided by these systems can be counterproductive in getting patient to understand their training.

Home-based BF with training devices has been proposed in the past [14-17]. Studies have demonstrated equivalent efficacy to office-based therapy while being more cost-effective. However, due to technical limitations, these past devices do not have the capabilities for therapists to remotely monitor and track patient compliance, which have significantly limited their adoption into clinical practice.

We have developed a new Internet-of-Medical-Things to deliver home-based BF. The system consists of an insertable probe with sensors to collect data on anal sphincter muscle contractility and rectal lumen pressures. The probe is wirelessly connected to a mobile app to provide real-time visual feedback of muscle activities to the user. The app records and processes training session data and clinical indicators (symptoms & bowel diary) from user input. Processed data will be sent to a cloud server to allow health providers to track progress and aid in decision-making. The proposed device will allow for instrumented anorectal BF training to be performed by patients at home.

**3. AIMS/OBJECTIVES/HYPOTHESES**

*Aim*

To compare the treatment efficacy of anorectal biofeedback (clinical indicators and physiological parameters) performed with a novel home-based device with conventional therapy options in patients with functional defecation disorders.

*Faecal incontinence is a heterogeneous condition, there is no single parameter to adequately define or represent an optimal way of assessing clinical outcomes. A range of subjective and physiological measures of bowel function are used as outcomes measures.*

*Site 1 Specific* - A feasibility assessment at Site 1 will evaluate the accuracy and repeatability of the prototype (V2) against anorectal manometry*

1. *Evaluate the capability of experimental device to generate anorectal exercise profiles*
2. *Compare the accuracy of experimental device against anorectal manometry*

*Site 1 and Site 2*

*Subjective primary outcome measures*

1. *Vaizey and Wexner faecal incontinence scores*
2. *Faecal incontinent episodes (days per week)*

*Objective primary outcome measure*

1. *Anal sphincter squeeze pressure (mmHg)*

*Secondary outcome measures*

1. *Anal sphincter resting pressure (mmHg)*
2. *Patient’s own view of effectiveness rating (“worse”, “same”, “improved”, or “cured”) and rating of that change in an ordinal scale of – 5 to + 5*
3. *Frequency of bowel motions and symptoms*
4. *[Experimental device group] Usability interview – 1. Ease of use, 2. Clarity of instructions, 3. Ease of cleaning, 4. Any additional Issues*

Hypotheses

We hypothesised that instrumented home BF therapy with the novel device is as efficacious as clinic-based BF therapy

**4. PARTICIPATING SITES**

1. GI Motility Clinic, Camden Hospital, NSW, 2570
2. Advanced GI Surgery Unit, Royal Prince Alfred Hospital, NSW, 2050

**5. RESEARCH PLAN/STUDY DESIGN**

Randomised controlled trial – Phase 0/Pilot study

**5.1 Type of study:** Prospective clinical research

**5.2 Expected study duration:** The start time is anticipated to be 01 April 2022 with completion of the project expected to be 31/3/2024. The length of the study is expected to last for 3 years.

**5.3 Data source and Population**

*Study population and size*

The Site 1 feasibility study will be a pilot study involving n=15 (n=20 with 33% dropout) participants per group. This study will evaluate the capability of the experimental device to provide instrumented BF (create distinct BF exercise profiles and is understood by the user) and compare the data against anorectal manometry. This is similar to our previous study [18] but will use the new prototype (V2) and be in a patient population.

The data from this site will not be analysed together with data from Site 2, given the differences in methodology. However, data from Site 1 will be used to improve the statistical analysis plan for Site 2. Currently, the power calculations for Site 2 are based on a previous study [19], but data from Site 1 will be integrated to improve the calculation of sample size and power for Site 2.

Site 2 sample size and power calculations

Based on a previous study of biofeedback treatment for faecal incontinence [19], we estimate that for assessing home-BF and clinic-BF efficacy with a sample size of 30 per group (n=40 with 33% dropout), the paired t-test at the 0.05 significance level can detect a mean change in anorectal function measure of at least 0.45 SD with 0.80 power. This corresponds to at least a mean decrease of 8 in Vaizey/Wexner faecal incontinence score, decrease of 0.6 in faecal incontinence episodes, and 40% increase in anal sphincter squeeze pressure.

For the non-inferiority study, based on changes in the primary outcome measures, with n=30 per study arm, the test for non-inferiority has 0.70 power to reject, at the 0.05 significance level, the null hypothesis that the mean change due to home-BF is smaller than clinic-BF by at least 0.44 SD in favour of he alternative hypothesis that mean changes due to home-BF is no more than 0.44 SD lower than clinic-BF (i.e. Ho: ΔHB-ΔOB<−0.44SD vs. Ha: ΔHB-ΔOB≤−0.44SD).

Using estimates from previous study [19], we consider home-BF no worse than or better than clinic-BF if the home-BF group had at least 9 point decrease in Vaizey/Wexner faecal incontinence score (11 for clinic-BF, SD=3), 0.95 days/week decrease in faecal incontinence episodes (1.1 for clinic-BF, SD=0.25), and 19 mmHg increase in anal sphincter squeeze pressure (25 mmHg for clinic-BF, SD=10).

*Data collection/Outcome measurements*

**Demographics and background history**

- Age, gender, height, weight, bowel history and symptoms, defection disorder diagnosis

**Anorectal manometry measurements**

- Manometry measurements before and after therapy: anal sphincter tone during rest and squeeze, rectal pressure during rest and push manoeuvre, anorectal muscle coordination during push manoeuvre.

**During biofeedback therapy**

- Participants keep a physical or digital bowel diary (bowel habits, stool consistency, symptoms)
- Participants keep a physical or digital food diary (meals, macro nutrients consumes, fibre consumed)
- The Vaizey and Wexner faecal incontinence score completed at the first and last appointment [20, 21]
- A the last appointment, participant provide self-evaluation of treatment efficacy and change [22]

**5.4 Recruitment and Screening**

Patients referred to study sites for BF therapy will be asked to take part in this study. The PIs at their respective sites (Dr Ho and Dr Ng) will determine eligibility. Potential participants are provided with the information sheet and consent form one week before their appointment as part of the standard preparation pack for patients. On the day of the test, Dr Zhou or Dr Ng (not involved in patient’s treatment) will engage with the potential participants by going through the patient information sheet with them. During this process the investigator will ensure the potential participant understands the study and risks, answer any questions, and, aware of the risks and how their data will be used.

**5.5 Inclusion criteria**

- Males and females 18 to 65 years
- Diagnosed with urge faecal incontinence or faecal seepage
- Able to provide verbal and written consent

**5.6 Exclusion criteria**

- Pregnant women and the human foetus
- People under the age of 18
- People with a cognitive impairment, an intellectual disability or a mental illness
- Anorectal surgery within the last 3 years
- Presence of active anal fissure and/or symptomatic haemorrhoids
- Individuals with implantable or portable electro-mechanical medical devices (e.g. pacemaker, sacral nerve stimulation impact)

**5.7 Consent Process**

Dr Ho (site 1) and Dr Ng (site 2) will obtain written consent from eligible participants or delegate the duty to Dr Zhou (site 1 and 2). Consent will be sought on the day of their study after potential participants have been give project information and opportunity to discuss the study with an investigator. It will be made clear that participation is voluntary and that the participant may withdraw at any stage with no detriment to their health care. Investigator will gauge their understanding by asking the potential participants questions about what they believe they are being asked to do. Investigators will also address any questions that arise from the participants in regards to the study. If the participants have any concerns or questions that arise at any stage in the future, they may contact Dr Zhou to further discuss them. Formal consent will be obtained in writing immediately following this as the information sheet and consent form is read by/to the participant, who may then indicate their voluntary participation by signing the consent form. A copy of the consent and information sheet will be provided to the participant to keep.

Participants may wish to withdraw from the study at any time. In the event the participant wishes to withdraw from the study, we will retain all data collected up to the point with withdraw.

**5.8 Study Procedure**

Patients arrive at a study site to undergo routine diagnostic anorectal manometry. If a patient is diagnosed with a functional faecal incontinence and suitable for BF therapy they will be asked to take part in this study. Randomization used permuted blocks of 4 with 1:1 assignment into the parallel study groups described in Section 5.3. Random numbers generated in advance and placed into sequentially numbered opaque envelopes, sealed and used for subject assignment. After screening, and once patients met the study inclusion and exclusion criteria, patients are enrolled into one of the treatment arms, by opening the sealed envelope. The therapist (Dr Patton or Mrs McHutchison) and patient are not blinded to the treatment.

The specialist nurse therapist provides advice regarding bowel habits, exercise, laxatives, dietary fibre and fluid intake, and timed-toilet training during the initial consultation to all participants. The therapist will teach participants how to improve their push effort by using postural and diaphragmatic breathing techniques and instruct them to practice these manoeuvres at home for 15 minutes, three times a day.

*Clinic-based biofeedback using anorectal manometry*

In addition to the aforementioned general instructions, participants receiving clinic-based BF (**Group A**) will be attending a maximum of six in-clinic therapy sessions over 2 months. BF training uses the anorectal manometry. Manometry catheters have several circumferential pressure sensors that straddle the entire anal canal and a proximal sensor placed in the rectum, providing continuous and dynamic spatiotemporal mapping of anorectal pressures. During anorectal BF therapy, a sheath is placed over the catheter and inserted into the anal canal. The system and therapist provides provide visual/audio feedback to the patient and record exercise parameters to establish baseline or evaluate progress. The key BF manoeuvres are:

1. **Rest:** after probe placement, a period of time is given for the patient to relax such that anal sphincter tone returns to basal levels. Anal resting pressure is measured using the averaged resting pressure over the length of the anal canal over a period of 20 seconds. This pressure is a composition of the internal and external anal sphincter and, to a lesser extent, by the hemorrhoidal plexus.
2. **Squeeze and hold:** The patient is asked to squeeze the anus for as long as possible, for a maximum of 30 seconds. By convention, this manoeuvre is performed three times. Both the maximal contractile pressure and endurance times are measured. Ideally, rectal pressure should not increase, because that would imply the patient has contracted the abdominal wall.
3. **Simulated defecation (push):** The patient bear down as if to defecation and attempt to expel the catheter. This manoeuvre should produce sufficient rectal propulsion pressures with simultaneous anal sphincter relaxation (>20% relaxation).

During treatment period, patients are asked to keep a bowel and food diary. During their in-clinic visits, the therapist will provide consultation on the patient’s progress and any changes to clinical indicators.

*Home-based biofeedback using new device*

In addition to the general instructions, patients selected for home-based BF with novel device (**Group B**) will be trained on the use of home-training device by the therapist. For feasibility study at Site 1, participants will use the device in the clinic under supervision for a maximum of six sessions.

Accuracy and repeatability of prototype V2 will be assessed in the first five (n=5) group B participants. Participant manometry parameters (e.g. resting, push, squeeze) will be compared against the prototype data during their first training session. Capabilities of the V2 sensors will compared against previous V1 data [18] to determine if the V2 prototype is suitable for use. If V2 sensors are not deemed suitable, the trial will be temporary suspended while the technical issues are resolved. During this time, the participant will be removed from the study and begin conventional treatment. If V2 sensors are suitable the participant will continue with trial and subsequent sessions.

Group B participants will undergo 6 supervised clinic sessions with the experimental device. After session two, they may also take the device home and continue training during the treatment period. The wireless device contains sensors to measure anal sphincter and rectal pressures. A condom sheath is placed over the probe before it is inserted into the anal canal. The user will download the training program (mobile app) onto their mobile phones, which will guide the user through the required exercises (identical to the clinic-based BF manoeuvres listed above). Participants will be asked to insert the probe and perform training for 5 – 10 minutes daily over a training period of 2 months. Training data is send to a cloud server for therapist to monitor compliance and correct usage. The participants are asked to keep daily bowel and food diaries on the app, this information is sent to the cloud accessible only by the therapist and investigators.

Upon completion of the feasibility study, home-based BF with novel device at Site 2 (Group B) will only undergo one supervised training session with the novel device. Participants then take the device home and continue training unsupervised for the duration of their treatment period. The therapist monitor adhesion remotely through the cloud server and will arrange fortnightly telehealth consultations to provide encouragement and evaluate progress.

At the end of the treatment period, participants in Group B will undergo a short useability interview. The therapist will ask the participant questions regarding the 1. Ease of use, 2. Clarity of instructions, 3. Ease of cleaning, 4. Any additional Issues.

*Data analyses*

At the end of the treatment period participants will return to the clinic for a repeat anorectal manometry study to determine changes in anorectal physiology parameters. The therapist will also review their bowel and food diaries. The therapist will discuss the results with the participants and plan the next course of action.

**Site 1 specific analysis***

A comparison between manometry and experimental device parameters will be assessed to gauge prototype V2 accuracy and repeatability.

**Site 1 and 2 analysis**

The manometric parameters (maximum anal sphincter squeeze pressure (mmHg) and mean anal sphincter resting pressure (mmHg)) for the period before treatment would be compared against parameters after treatment. Clinical indicators Vaizey/Wexner faecal incontinence score, faecal incontinence episodes (days/week), frequency of bowel motions & symptoms, and patient’s own view of effectiveness rating will be analysed. Univariate analysis (paired t-test or Wilcoxon) will be used to evaluate manometric parameter changes, while chi square test will be used to compare clinical indicators.

Non-inferiority study analysis described in Section 5.3 will be used to determine if null hypothesis is accepted or rejected.

***
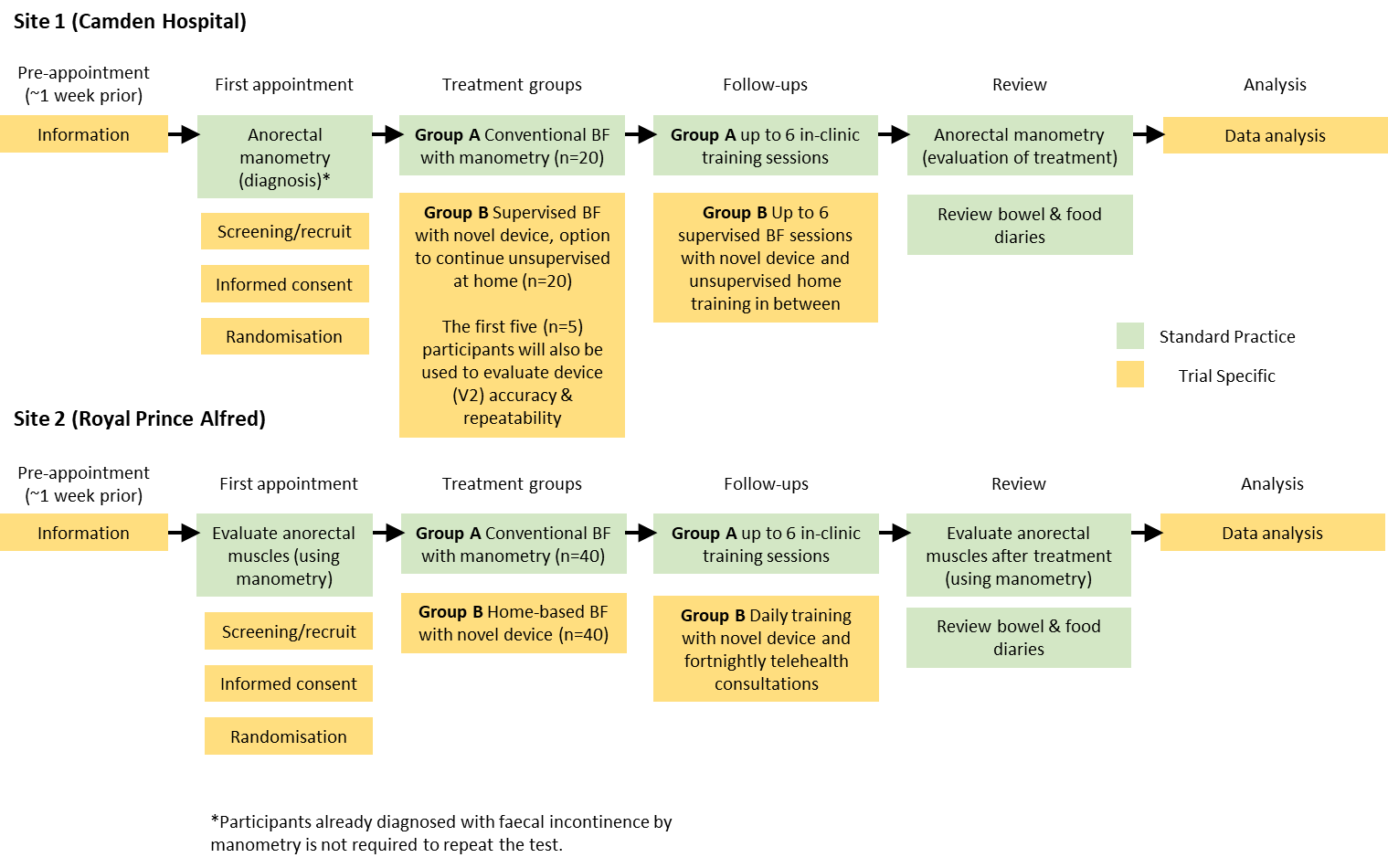
***

***Figure 3.*** *Summary of study procedure*

**7. ETHICAL CONSIDERATIONS**

**7.1 Study Procedure Benefits**

This study should provide a direct benefit to the participant in the form of anorectal biofeedback therapy. Participants in all study groups should receive some level of therapy equal to or higher than what is currently available. The outcomes of this study will increase the clinical community’s understanding defecation disorders and anorectal physiology. Our findings may contribute towards the development of a novel approach to deliver BF therapy. Ultimately this may, lead to readily accessible treatment of defection disorders. Data from this study will be used as part of regulatory submission and possible commercialisation of this product in Australia. This outcome will enable the hospitals to provide this intervention after the completion of the project.

**7.2 Study Procedure Risks**

Potential risks associated with the novel home-based BF device are summarised in Table 2.

**Table 2.** Identified risks and mitigation activities

| Identified Risk | Probability | Impact | Mitigation Strategies |
| --- | --- | --- | --- |
| Bacterial and faecal contamination on probe | Occasional  (1 in 10K) | Minor | - Labelling: directions for use, single person use only - Condom sheath over probe for insertion - Wipe down with clean-wipes after use |
| Probe breaking during use | Remote  (1 in 100K) | Serious | - Bend testing of probe determined >5 kg of pressure required to compromise structural integrity (far above anorectal muscle forces). - Insertion and use in the lying position to avoid accidental slips and falls |
| Perform exercise incorrectly | Probably  (1 in 1K) | Negligible | - Training with therapist (first session) before home use - Software to detect incorrect placement or use, then instructs user to adjust or remove probe |
| Anal tearing/perforation | Remote  (1 in 100K) | Serious | - Exclude users with anal fissure and haemorrhoids - Use with lubricant - Immediate removal with any discomfort or pain |
| Electric shock | Remote  (1 in 100K) | Minor | - Power source (battery) is located in the base and external of the body during use - Labelling: not to be submerged in water - In-built moisture detection kill switch in circuit - Low voltage lithium battery (3.7 V; 700 mAh) |

Precautions have been taken to mitigate all risks but in the chance of an adverse event the participant can contact the lead investigator (Dr Jerry Zhou). Dr Zhou will ask the participant to stop using the device and discuss the severity of the incident with study clinicians (Dr Ho or Dr Ng) to best advise participant of their course of action (e.g. return the device for replacement, consultation with clinician, visit emergency department).

The recording and reporting of adverse events will be classified based on their severity (“Negligible”, “Minor”, “Serious”, “Critical”, and “Catastrophic”). All events classified as “minor” or above in severity will be recorded and notified to the Principle investigator (Dr Ho) and site coordinating investigator (Dr Ng). Events classified as “Serious” or above will be will reported to HREA in to form of a “safety report” and trial will be temporarily suspended to investigate cause of event and implement appropriate mitigation strategies (e.g. instructions-of-use, technical updates, update cleaning procedure).

The clinicians involved in this study (Dr Ho and Dr Ng) will be available to help if any adverse events or symptoms occurs during the study. They will be able to provide medical advice and determine the best course of action.

To avoid coercion or inducement, an investigator not directly involved in the participant’s treatment (e.g. Dr Jerry Zhou) will be explaining the procedure and obtaining informed consent.

**7.2.1 COVID-19 Considerations**

The in-person consultation and BF training carry a low risk of aerosolisation of virus particles and transmission to the staff. The study will follow general contact and droplet precautions detailed in the advice from: https://www.cec.health.nsw.gov.au/keep-patients-safe/COVID-19. Investigator will provide their own protective personal protective wear (gloves and mask), which will be wore when approaching participants and instructing the procedure. Investigators will also practice social distancing and maintain a 1.5m distance when conversing with participants. Participants will be asked if they experience COVID-19/flu-like symptoms, in the case of a positive symptoms, they would not be asked to take part in this study. The home-based devices are single person use and does not pose a risk of cross transmission of virus particles between participants. The room and equipment (bed, table, handles etc.) will be thoroughly cleaned with disinfectant between participants.

**7.3 Confidentiality and Privacy**

Identifiable participant information will remain on site on the Hospital’s password protected computer. Identifiable information (name, MRN, date of birth) will be kept on a “decoding sheet” in a separate folder to study data, only the PI will have access to the decoding sheet. De-identified health data will be stored on the site network using a data management software (RedCap).

Data collected by the device and app will be sent to a secure cloud server managed internally by Western Sydney University and only accessible by study investigators. This data used entered into RedCap in a non-identifiable form for analysis by investigators. Dissemination of data will in the form of descriptive statistics (e.g. mean±SD) in results and publications.

**7.4 Data storage and Record retention**

The information collected on paper copies of participant information will be physically locked up in a cabinet located in the offices of the study sites. Furthermore, identifiable electronic records will remain on the Hospital network folders. Device and app training data are stored on the secure Western Sydney University cloud server and the password is only accessible by the coordinating investigators and therapists.

A copy of the non-identifiable data for analysis will also be stored in RedCap a password protected computer within a locked office (30.2.65) at the School of Medicine, University of Western Sydney. As such only the investigators will have access to this information. The storage of data and information will be closely monitored by the principal investigators. If the confidentiality of participants is violated it will be immediately reported to a central monitoring committee. The information collected will be retained for 15 years per Section 2.3 Australian Code for the Responsible Conduct of Research. At the end of these 15 years paper copies will be shredded and appropriately disposed while electronic data will be deleted.

**8. CONFLICT OF INTEREST**

All investigators declare no financial interest or benefit that may arise from the direct applications of this research.

The investigators Dr Ho and Dr Zhou are inventors of the experimental device and Western Sydney University is the owners of the intellectual property (IP). The inventors and Western Sydney University may have a financial interest in the device if it was commercialised

**9. FUNDING**

This project is funded by a research grant from SPHERE (Maridulu Budyari Gumal) Frontiers Technology and Translational Gastroenterology Laboratory at School of Medicine, Western Sydney University.

**10. RESEARCH OUTCOMES**

The proposed research outcomes will contribute towards our understanding of BF therapy and anorectal physiological. The broader impact of our findings may lead to improvements in the delivery of BF therapy though a home-based device that provides instrumented BF therapy equivalent to clinic-based BF.

The results of this study will be disseminated at national and international conferences along with relevant journals (such as The American Journal of Gastroenterology). The results of the individual studies will be available each participant (if they are interested) and provide as a lay report. The investigators aim to publish the results in an open access journal so that participants and the public would be able to readily access the outcomes of this study.

**11. REFERECNES**

[1] S. S. Rao, A. E. Bharucha, G. Chiarioni, R. Felt-Bersma, C. Knowles, A. Malcolm, A. Wald, “Anorectal disorders,” Gas-troenterology, 150(6), pp.1430-1442. 2016.

[2] S. S. C. Rao, M. A Benninga, A. E. Bharucha, G. Chiarioni, C. Di Lorenzo, W. E. Whitehead, “ANMS‐ESNM position pa-per and consensus guidelines on biofeedback therapy for anorectal disorders,” Neurogastroenterol. Motil., 27(5), pp.594-609. 2015.

[3] S. S. C. Rao, A. K. Tuteja, T. Vellema, J. Kempf, M. Stessman, “Dyssynergic defecation: demographics, symptoms, stool patterns, and quality of life,” J. Clin. Gastroenterol, 38(8), pp.680-685. 2004.

[4] C. Pehl, H. Seidl, N. Scalercio, F. Gundling, T. Schmidt, W. Schepp, S. Labermeyer, “Accuracy of anorectal manometry in patients with fecal incontinence,” Digestion, 86(2), pp.78-85. 2012.

[5] K. S. Ng, Y. Sivakumaran, N. Nassar, M. A. Gladman, “Fecal incontinence: community prevalence and associated fac-tors—a systematic review,” Dis. Colon Rectum, 58(12), pp.1194-1209. 2015.

[6] M. Camilleri, A. C. Ford, G. M. Mawe, P. G. Dinning, S. S. C. Rao, W. D. Chey, M. Simrén, A. Lembo, T. M. Young-Fadok, L. Chang, “Chronic constipation,” Nat. Rev. Dis. Primers, 3(1), pp.1-19. 2017.

[7] S. S. C. Rao, M. A Benninga, A. E. Bharucha, G. Chiarioni, C. Di Lorenzo, W. E. Whitehead, “ANMS‐ESNM position pa-per and consensus guidelines on biofeedback therapy for anorectal disorders,” Neurogastroenterol. Motil., 27(5), pp.594-609. 2015.

[8] S. S. C. Rao, K. Seaton, M. Miller, K. Brown, I. Nygaard, P. Stumbo, B. Zimmerman, K. Schulze, “Randomized controlled trial of biofeedback, sham feedback, and standard therapy for dyssynergic defecation,” Clin. Gastroenterol. Hepatol., 5(3), pp.331-338. 2007.

[9] S. Heymen, Y. Scarlett, K. Jones, Y. Ringel, D. Drossman, W. E. Whitehead, “Randomized controlled trial shows bio-feedback to be superior to pelvic floor exercises for fecal incontinence,” Dis. Colon Rectum, 52(10), pp.1730-1737. 2009.

[10] C. Norton, M. A. Kamm, “Anal sphincter biofeedback and pelvic floor exercises for faecal incontinence in adults—a systematic review,” Aliment. Pharmacol. Ther., 2001. 15(8): p. 1147-54. 2001.

[11] S. S. C. Rao, F. Azpiroz, N. Diamant, P. Enck, G. Tougas, A. Wald, “Minimum standards of anorectal manometry,” Neurogastroenterol. Motil., 14(5), pp.553-559. 2002.

[12] F. Azpiroz, P. Enck, W.E. Whitehead, “Anorectal functional testing: review of collective experience,” Am. J. Gastroen-terol., 97(2): p. 232-40. 2002.

[13] T. H. Lee, A. E. Bharucha, “How to Perform and Interpret a High-resolution Anorectal Manometry Test,” J. Neurogas-troenterol. Motil., 22(1): p. 46-59. 2016.

[14] S. S. C. Rao, J. T. Go, J. Valestin, J. Schneider, “Home Biofeedback for the Treatment of Dyssynergic Defecation: Does It Improve Quality of Life and Is It Cost-Effective?” Am. J. Gastroenterol., 2019. 114(6): p. 938-944. 2019.

[15] S. S. C. Rao, J. A. Valestin, X. Xiang, S. Hamdy, C. S. Bradley, M. B. Zimmerman, “Home-based versus office-based bi-ofeedback therapy for constipation with dyssynergic defecation: a randomised controlled trial,” Lancet Gastroenterol. Hepatol., 3(11), pp.768-777. 2018.

[16] D. C. Damin, F. Hommerding, D. Schirmer, P. R. Sanches, D. P. S. Junior, A. F. Müller, P. R. Thome “Patient-controlled biofeedback device for the treatment of fecal incontinence: a pilot study,” Appl. Psychophysiol. Biofeedback, 42(2), pp.133-137. 2017.

[17] A. Sharma, X. Xiang, Y. Yan, T. Patcharatrakul, R. Parr, S. S. Rao, “Home Biofeedback Therapy Improves Fecal Incontinence Severity and Quality of Life in a Non-Inferiority Randomized Controlled Study: 416,” Am. J. Gastroenterol. ACG, 113, p.S246. 2018

[18] Zhou, J., Ho, V. and Javadi, B., 2022. New Internet of Medical Things for Home-Based Treatment of Anorectal Disorders. Sensors, 22(2), p.625.

[19] Heymen, S., Scarlett, Y., Jones, K., Ringel, Y., Drossman, D. and Whitehead, W.E., 2009. Randomized controlled trial shows biofeedback to be superior to pelvic floor exercises for fecal incontinence. Diseases of the Colon & Rectum, 52(10), pp.1730-1737.

[20] Jorge, J.M.N. and Wexner, S.D., 1993. Etiology and management of fecal incontinence. Diseases of the colon & rectum, 36(1), pp.77-97.

[21] Bols, E.M.J., Hendriks, H.J.M., Berghmans, L.C.M., Baeten, C.G.M.I. and De Bie, R.A., 2013. Responsiveness and interpretability of incontinence severity scores and FIQL in patients with fecal incontinence: a secondary analysis from a randomized controlled trial. International urogynecology journal, 24(3), pp.469-478.

[22] Norton, C., Chelvanayagam, S., Wilson-Barnett, J., Redfern, S. and Kamm, M.A., 2003. Randomized controlled trial of biofeedback for fecal incontinence. Gastroenterology, 125(5), pp.1320-1329.
