# Supplementary material for: Randomized controlled trial of an Internet-of-Medical-Things device for patient-guided anorectal biofeedback therapy
Source: PLoS One. 2024 Sep 27;19(9):e0311134. doi: 10.1371/journal.pone.0311134 (PMC11432836; doi:10.1371/journal.pone.0311134)
Supplement: Attachment — Submitted filename: ResponsetoReviewers_19MAY24.docx [file pone.0311134.s003.docx]

Dr Jerry Zhou

Senior Lecturer

School of Medicine, Western Sydney University

19 April 2024

Editorial Board

PLOS ONE

Dear Editorial Board and Reviewers,

We would like to thank the Editorial Board and Reviewers for their time and feedback on our manuscript [PONE-D-24-06266R1].

Please find below our response to reviewer comments:

**Comments to the Author**

1. If the authors have adequately addressed your comments raised in a previous round of review and you feel that this manuscript is now acceptable for publication, you may indicate that here to bypass the “Comments to the Author” section, enter your conflict of interest statement in the “Confidential to Editor” section, and submit your "Accept" recommendation.

Reviewer #1: All comments have been addressed

Reviewer #2: (No Response)

Reviewer #3: All comments have been addressed

2. Is the manuscript technically sound, and do the data support the conclusions?

The manuscript must describe a technically sound piece of scientific research with data that supports the conclusions. Experiments must have been conducted rigorously, with appropriate controls, replication, and sample sizes. The conclusions must be drawn appropriately based on the data presented.

Reviewer #1: (No Response)

Reviewer #2: Partly

Reviewer #3: Yes

3. Has the statistical analysis been performed appropriately and rigorously?

Reviewer #1: (No Response)

Reviewer #2: No

Reviewer #3: Yes

4. Have the authors made all data underlying the findings in their manuscript fully available?

The PLOS Data policy requires authors to make all data underlying the findings described in their manuscript fully available without restriction, with rare exception (please refer to the Data Availability Statement in the manuscript PDF file). The data should be provided as part of the manuscript or its supporting information, or deposited to a public repository. For example, in addition to summary statistics, the data points behind means, medians and variance measures should be available. If there are restrictions on publicly sharing data—e.g. participant privacy or use of data from a third party—those must be specified.

Reviewer #1: (No Response)

Reviewer #2: No

Reviewer #3: Yes

**Data for this study has been made available on FigShare via the following link**

**https://doi.org/10.6084/m9.figshare.25844506.v1**

5. Is the manuscript presented in an intelligible fashion and written in standard English?

PLOS ONE does not copyedit accepted manuscripts, so the language in submitted articles must be clear, correct, and unambiguous. Any typographical or grammatical errors should be corrected at revision, so please note any specific errors here.

Reviewer #1: (No Response)

Reviewer #2: Yes

Reviewer #3: Yes

6. Review Comments to the Author

Please use the space provided to explain your answers to the questions above. You may also include additional comments for the author, including concerns about dual publication, research ethics, or publication ethics. (Please upload your review as an attachment if it exceeds 20,000 characters)

Reviewer #1: (No Response)

Reviewer #2: (No Response)

Reviewer #3: The authors have adequately addressed the comments raised in a previous round of review. However, I have one more question about the compliance of the experimental device. How many hour per week in average that the participants use the device for training? Is that significantly different from the standard device? We hope you find our manuscript suitable for publication.

**Both, the experimental device and standard device were used in the clinic, the difference being that patients were self-guided when using the experimental device but nurse-guided for the standard device. No issue with patient compliance were observed. Technical issues (e.g. phone/device pairing) were addressed by the supervising specialized nurse.**

Kind regards,

Dr Jerry Zhou
