# Supplementary material for: Randomized controlled trial of an Internet-of-Medical-Things device for patient-guided anorectal biofeedback therapy
Source: PLoS One. 2024 Sep 27;19(9):e0311134. doi: 10.1371/journal.pone.0311134 (PMC11432836; doi:10.1371/journal.pone.0311134)
Supplement: Attachment — Submitted filename: ResponsetoReviewers_23JUL24.docx [file pone.0311134.s004.docx]

Dr Jerry Zhou

Senior Lecturer

School of Medicine, Western Sydney University

23 July 2024

Editorial Board

PLOS ONE

Dear Editorial Board and Reviewers,

We would like to thank the Editorial Board and Reviewers for their time and feedback on our manuscript [PONE-D-24-06266R1].

Please find below our response to reviewer comments:

**Review Comments to the Author**

Reviewer #3: All questions have been answered. I accept this manuscript for publication. This manucript will be very useful to the readers.

We would like to thank Reviewer #3 for their time and feedback to improving our manuscript.

Reviewer #4: This is a proof of concept study of a new device "Internet of Medical Things",. The authors have thought through and developed a pressure-based probe system that provides biofeedback, and have performed a careful study of their intended population and thoughtful statistical analysis including both study arm specific and between study arms. I have several concerns outlined below that should be addressed:

We would like to thank Reviewer #4 for their time and feedback to improving our manuscript.

1. The authors have chosen a truly bizarre and strange name for a medical device purported to be used for anorectal biofeedback. To the best of my reading the device has nothing to do with INTERNET and I personally feel that unintentionally it either trivializes or makes it sound "game like" (MEDICAL THINGS) for a respectable, proven and somewhat sensitive and intimate training-biofeedback therapy for anorectal disorders. The authors should consider changing the name of the device especially for reporting their research in medical literature.

The proposed commercial name for this medical device is yet to be determined but would be inline with the reviewer’s expectation of a medical device for biofeedback therapy. The name in the title “Internet-of-Medical-Things device” describes the device’s capabilities and to differentiate it from other non-connected biofeedback devices. The proposed device utilises the Internet-of-Things (IoT) concept to healthcare, where a medical device is connected to a network to allow for remote monitoring by health providers.

2. The authors say that the patients using the new device were self-guided, but the nurse therapist, albeit a different nurse than the nurse performing standard ARM-assisted biofeedback was present and assisted the patients throughout the study. It is unclear how much assistance they provided to the patient compared to ARM-guided, how much time this person spent with the patient, and any other information on this aspect of biofeedback, as the authors should provide more details and explicit information.

Additional information has been provided in **Methods: Biofeedback therapy and assessment**.

[Line 112] “*The clinical nurse provide a brief tutorial on how to setup and use the device. During the first biofeedback session, the nurse provided guidance by showing patients the correct positioning of the device, app operation, and how to correctly perform exercise maneuvers. Up to 6 follow-up sessions were provided, involving a 30 minute review session with the nurse on their bowel and diet habits, and a self-guided biofeedback session. In the follow-up biofeedback sessions, patients were asked to perform biofeedback by themselves by following the instructions on the app. The nurse was only allowed to assist in events of technical issues or intervene if nurse felt the patient was at risk of injury.”*

3. The authors say in discussion that " patient's literacy did not appear to impact their ability to use the device". How do they know this? What is the evidence? Did they collect any information on user friendliness of device or device tolerability? If so this must be provided and substantiated, if not this statement should be removed.

Usability was assessed based on patient’s self-described capabilities. This is described in **Results: Usability**

“*Participants self-described their technology literacy as “moderate to poor” but were all able to operate the system unguided after one training session with a therapist. When asked about potential use of the device at home, all participants were confident in their ability to use the system unsupervised and by themselves*.”

4. The authors mention that patients had to hold the device and at the same time pay attention to the biofeedback instructions, and this can be tough for a patient. How was the monitor or phone device held, and how close. Did patients have concerns about this? Please provide information?

There was only one incidence where a patient had to hold the device due to a very weak anal sphincter muscle. In this case, the phone was place on a stand on the bed where she was laying (approximately 20cm from her face). The patient did not report any issues in operating the phone and did not require assistance from the supervising nurse.

5. The accepted clinical outcome measure for evaluating fecal incontinence is 50% reduction in FI episodes, and is now used in all FI clinical trials. The authors should provide this information for this in both arms. Also they should report baseline and post treatment, number of bowel movements and number of FI episodes.

The frequency of bowel movements and number of FI episodes at baseline and post treatment has been amended in Table 2. Additionally, the clinical significance of a 50% reduction in FI episodes was added to Table 2 footnote.

6. Was investigator performing ARM measurements blinded to the baseline and arm of the study? Please provide.

No, the same nurse that performed the ARM measurements also conducted the biofeedback training for the respective study arm. The nurses were blinded to the baseline and post treatment ARM values.

7. Was Anal squeeze pressure, the maximum pressure generated or sustained pressure generated?

Maximum pressure generated. This has been updated in Table 2.

8. Was this nurse-assisted, self-guided treatment program or a self-guided treatment program? Please elaborate.

The device requires an initial training session from a healthcare provider (e.g. nurse). Following sessions are self-guided treatment program.

9. Reviewing Figure, 2 D, "Quick Sq exercise" When you are ready press button .....", how were patients able to do this , and hold probe in one hand and hold phone in one hand? Please explain. Also the letters were too small and problematic reading.

The phone would be held in a stand during biofeedback sessions. During the trial, most patients had both hands free to operate the app. One patient with very weak sphincter tone did need to hold the device while operating the phone, but had not issues operating with one hand.

During the trial, the phone is usually 20cm away from the patient. The text has not been an issue but we are away some elderly patient may have difficulty read the text and will increase the size.

10. Please explain and provide rationale for why passive incontinence patients were excluded as this is usually not the case in FI studies.

The mechanism for passive incontinence often involves impaired neurological signalling and/or hyposensitivity (such as stroke, nerve injury). This type of incontinence does not response well to biofeedback therapy and would be recommended nerve stimulation or surgery.

11.Abstract mentions safety and tolerability, and although I dont perceive any concerns with this kind of device, no information is provided regarding this part of study. Please provide.

Safety results was presented in Results: Safety.

12. In line 90, Fig 2 is mislabeled should be Fig 1

Amended in text.

13. Capitalization in line 166, typographical errors in line 192 and 268, please fix.

Amended in text.

14. Were there any problems with communication between the device and probe and how were they overcome? Probe retention inside the body could be a challenge?

There were no communication issues between the device and probe sensors, which were directly connected to the processor chip embedded in the device body. The device also had no communication issues with the mobile app. The Bluetooth module was in the device body and remained external during biofeedback.

There was no issues with probe retention inside the body as the device body was flared to prevent accidental insertion of the whole device.

Reviewer #5: PONE-D-24-06266R2 :

Thanks to the author for this well written manuscript. They performed a randomized controlled trial to assess a new device for biofeedback therapy in fecal incontinence.
The purpose of the study is very interesting because even if BF therapy is a first line treatment for FI, It is a real challenge for patient to find therapist which perform it. However, even the protocol is well described and conducted, there is some limitations.
Firstable, this a small sample size and results should be inetrepreted with precaution but this is a well conducted study.

We would like to thank Reviewer #5 for their time and feedback to improving our manuscript.

Secondly, there is a potential bias in the study population. All patients were referred to the care center for HD ARM. Did the authors know their previous FI management? As there is a wide majority of women, did they perform conventional BF before the study (and how many time before)? If possible, it would be interesting if the device show a non inferiority for a first line BF therapy.
Please describe more precisely the usability section (cf comments) and difficulties encountered by patients


Abstract:
No comment

Introduction:
No comment
Topic of the study is well described and placed. The objective is well described too

Methods:
I think that non inclusion criteria are too strict and probably lead to the small sample size…More over, patients with diarrhea or Bristol 6 or more and who are incontinent still perform “standard biofeedback therapy” and maybe should not be excluded from this protocol. Same comment for opioids and antidepressant.

Line 110-114: Authors described the difference between the 2 arms of the protocol and there is a potential biais, especially for Arm A. Indeed, nurse provided encouragement for patients and it could have an impact on HD ARM results (better with motivation)
Same protocol for both arm would be preferable.

Results:
Please add population description in this section instead of the first part of the discussion.

How were evaluate patients for usability? Likert scale or numeric scale? Other?
Line 215: “some patients” : please describe preciselyhow many patients because there is a small sample size and if “some” correspond to 5 or more, it could be interpreted as 25%...
Please expand why they reported their companion app as confusing

Please add, if avaiblable, how many training sessions performed the patient with the medical device. Did they use it every day? Once a week (same intervention than the conventionnal group)? These results could demonstarte the usability and the adherence to the device but also help readers to interpret the second section of the results. Indeed, one group had a weekly sessions vs a daily session, which may lead to better results for the daily intervention? Please justify

Authors did not explore constipation or did not describe it in their manuscript. However, both symptoms coexists for patients and constipation and its management can improve FI. Please add a paragraph if possible (results or discussion section).

All patients had severe symptoms and improved to moderate symptoms and both therapies showed improvement. I think that the use of the PGI-I (patient global impression of improvement) rather than a 10-point Likert scale (-5 to +5) is a better way to assess the improvement.

Discussion:
No comment
Thanks to the author for their correct conclusion (as safe and usable device) and not for non-inferiority therapy.

Kind regards,

Dr Jerry Zhou
